# Egypt’s Initial Experience With Robotic-Assisted Cystogastrostomy and Pancreatic Debridement for Large Walled-Off Pancreatic Necrosis: A Report of Two Cases

**DOI:** 10.7759/cureus.32005

**Published:** 2022-11-29

**Authors:** Mohammad A Abd-erRazik, Mohamed A Abdel Hamid, Amier M Rashed

**Affiliations:** 1 Department of General Surgery, Ain Shams University, Cairo, EGY

**Keywords:** robotic-assisted surgery, acute necrotic pancreatic collection, wopn, robotic cystogastrostomy, robotic pancreatic surgery

## Abstract

Gallstones are the most common cause of acute pancreatitis (AP). Walled-off pancreatic necrosis (WOPN) is one of the sequelae of AP. Endoscopic and laparoscopic techniques for cystogastrostomy have been reported in the literature as treatment options for complicated or symptomatic WOPN. Here, we describe two cases of gallstone-related AP complicated by WOPN treated by robotic-assisted transgastric cystogastrostomy and cholecystectomy.

## Introduction

Acute pancreatitis (AP) is an acute inflammatory condition of the pancreas. Gallstones are the most common cause of AP [1]. Other causes include alcohol abuse, hypertriglyceridemia, toxins, drug-induced, mechanical/obstructive (pancreas divisum, annular pancreas, pancreatic head tumor), iatrogenic, infectious, hereditary, traumatic, and vascular; in 25% of the cases, it is idiopathic [2-6]. The incidence of AP is increasing globally. It is one of the most common gastrointestinal reasons for hospital admission [7].

Severe AP comprises about 25% of all pancreatitis patients. It is a life-threatening condition with an in-hospital mortality rate of around 15% [8]. A more severe form of AP is necrotizing pancreatitis, which occurs in 5-10% of patients [9]. The impact of pancreatic necrosis after AP may have severe consequences on patients’ quality of life that can persist for years or even for life [10].

Acute necrotic collection is a peripancreatic collection associated with pancreatic necrosis. It consists of fluid and necrotic tissue (of the pancreatic parenchyma and/or peripancreatic tissues). After weeks, it is usually called walled-off pancreatic necrosis (WOPN). WOPN is an encapsulated collection formed of pancreatic and/or peripancreatic necrosis with a well-defined, mature, inflammatory wall. Maturation usually occurs at least four weeks from the onset of AP [9,11].

## Case presentation

Here, we present the cases of two patients who underwent robotic-assisted cystogastrostomy and pancreatic debridement for WOPN. The first patient was a 52-year-old female patient who presented with epigastric pain radiating to the back, in addition to intolerance for oral intake for a month. She had a medical history of chronic liver disease secondary to hepatitis C virus infection. She underwent three cesarean sections. Five weeks before the presentation, she was admitted to another hospital with a clinical picture suggestive of acute gallstone pancreatitis and acute cholecystitis, which was managed conservatively. Her condition improved, and she was discharged two weeks later; however, vomiting, abdominal pain, and intolerance for oral intake continued. She lost 15 kg weight over a month.

**Figure 1:**
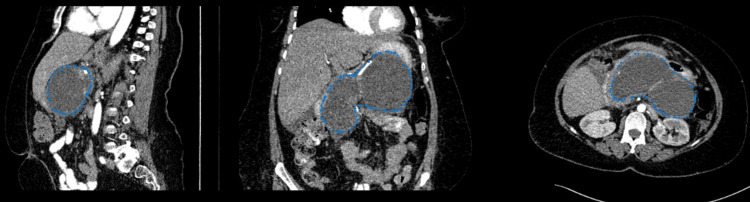
The first case CT scan showing the WOPN in three different planes (marked in blue). CT: computed tomography; WOPN: walled-off pancreatic necrosis

On admission to our hospital, the patient was vitally stable and fever-free. Her body mass index (BMI) was 38.3 kg/m^2^. She was incapable of lying flat due to the pain. Her abdominal examination revealed epigastric fullness and tenderness. Although there was a mild elevation in serum amylase and lipase levels, she had normal laboratory results. Abdominopelvic computed tomography (CT) scan with contrast (Figure 1) showed near-total replacement of the pancreas with a large loculated cyst measuring about 17 × 7.5 cm with a thin enhancing wall compressing and displacing the stomach anteriorly. The gallbladder was noted to have a thick wall and contained two tiny stones. No intra or extrahepatic biliary dilatations were noted on the scan.

The second patient was a 31-year-old male. He presented with abdominal pain and swelling with a history of acute severe pancreatitis post-endoscopic retrograde cholangiopancreatography (ERCP) for extraction of a common bile duct (CBD) stone. On admission, the patient had features of acute weight loss, with an obvious abdominal mass. His BMI was 20.5 kg/m^2^. He reported episodes of vomiting after meals which made him refuse oral intake. Apart from mild anemia and hypoalbuminemia, his lab results were within normal limits. Abdominopelvic CT scan showed two large collections, and the gallbladder was harboring multiple stones with a thick edematous wall, no intra or extrahepatic biliary radical dilatation, and a CBD stent in place. The first collection was in the lesser sac with a mature wall, and the second was an encapsulated subhepatic collection (related to the left lobe). We decided to insert an ultrasound-guided percutaneous draining catheter in the subhepatic collection a couple of days before surgery.

**Figure 2:**
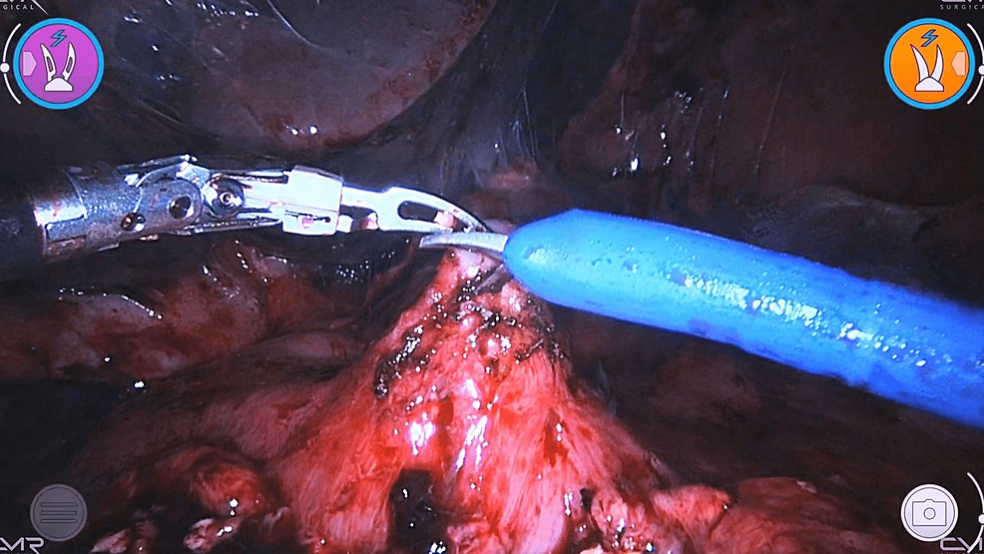
Anterior gastrotomy in case two.

**Figure 3:**
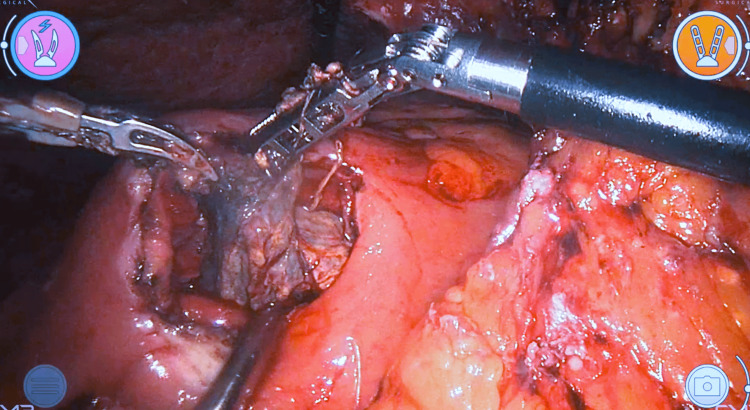
Pancreatic debridement in case one.

Both patients were scheduled for robotic-assisted laparoscopic cystogastrostomy and cholecystectomy. The surgical procedures were essentially the same for both patients. Patients were placed in the supine position and received general anesthesia. The open technique was used to insert the scope port. After acquiring sufficient pneumoperitoneum, the working ports were inserted under vision, two ports for the robotic instruments, and two for assistance. On exploration of the abdominal cavity, adhesions were noted and sharply cut. The subhepatic collection of the second patient was deroofed and cleaned. The stomach was identified as displaced anteriorly by the WOPN. An anterior gastrotomy was done (Figure 2). The posterior wall of the stomach was found stretched and elevated. A needle aspiration test was done to confirm the proximity of the cyst and exclude any vascular structures in the designated area of posterior gastrotomy, which showed turbid content. A 5 cm posterior gastrotomy was performed using scissors and monopolar cautery. Turbid fluid was suctioned (around 400 mL in the first and 150 mL in the second case), and necrotic tissue was removed from the cyst (necrosectomy) (Figure 3).

After irrigation and suction, the posterior gastric wall was sutured together with the WOPN using running 3-0 polydioxanone (PDS) sutures. For each patient, a nasogastric tube was placed in the stomach under vision. The anterior gastrotomy was also sutured using running 3-0 PDS sutures and an omental patch was placed.

Cholecystectomy was then started by obtaining the critical view of safety. The cystic duct and artery were identified, secured with metallic clips, and divided. Two intra-abdominal drains were placed in each case. Surgery time was around five hours in the first case and four hours in the second case. The blood loss was about 150 mL in the first case and 200 mL in the second case.

The first patient was transferred to the intensive care unit for a single day. She had an uncomplicated postoperative course apart from a single attack of fever of 38°C on the first postoperative day. She started oral intake on the fourth postoperative day. She was discharged on the sixth postoperative day. The patient’s symptoms dramatically improved. On the first postoperative day, her epigastric pain disappeared, and she was able to lie on her back without pain. On follow-up visits, the patient had no pain, and she regained tolerance for food with no nausea or vomiting.

The second patient was transferred directly from the operation theater to the ward. He started oral fluids on the third postoperative day and was discharged on the seventh postoperative day. All his symptoms improved, and he started to gain weight in the next follow-up visits. A month later, he suffered from epigastric pain and vomiting, which were controlled with a short course of proton-pump inhibitors and prokinetics.

## Discussion

This report presents two cases with WOPN, post-gallbladder stones related AP, treated with a robotic-assisted laparoscopic procedure.

For years, open surgical pancreatic debridement or necrosectomy was the standard of care for patients with WOPN or necrotizing pancreatitis [12]. However, the 90-day mortality rate of the procedure was reported to be 10.6%, on average, which increased with older age, the presence of comorbidities, and surgery within the first four weeks from the onset of pancreatic necrosis [13]. Laparoscopic management of pseudo-cyst of the pancreas or WOPN was reported in sporadic case presentations starting from the mid-90s [14]. Since then, it has gained more acceptance together with endoscopic drainage procedures. The main advantage of the laparoscopic over endoscopic procedure is the creation of a wide anastomosis between the cyst and the stomach without placing a stent [15].

Robotic cystogastrostomy was also reported for the management of WOPN in case reports [15,16] or small case series [17]. The robotic procedure has an array of advantages over the conventional laparoscopic procedure, starting with the magnification and the three-dimensional vision, which enhances the identification of the structures. Being located in the lesser sac, WOPN is challenging to tackle with the straight rigid conventional laparoscopic instruments; on the other hand, the articulating robotic instruments give more accurate movements, finer dissection, and firmer grip. The combination of the robotic-enhanced visualization system and instrument characteristics also results in more accurate, regular, and easier suturing results. Another advantage is better ergonomics for the surgeon, which decreases fatigue during surgery, potentially preventing wrong posture-related injuries. On the other hand, the relatively higher cost is the main challenge for the routine use of the robotic procedure. We secured the anastomosis with sutures instead of staplers, which can be used in such procedures [18]. This was done due to the lack of staplers as well as the advanced energy instruments in the robotic system we used. Other minor challenges such as choosing the right robotic bedside unit placement and port placement are considered to be temporary issues that will be resolved over time.

The overall experience with the robotic-assisted transgastric cystogastrostomy for the two cases, with relatively large WOPN, and one of these cases was morbidly obese, was very satisfactory. Even though they were chronologically considered to be the early cases done in our young robotic surgery program, the Ain Shams University Specialized Hospital robotic program only started on October 31, 2021. It is worth mentioning that, on reviewing reports from the manufacturing company of the robotic system used in those cases and by reviewing literature from Egypt, we did not find any previous similar cases. This makes these two procedures the first robotic-assisted cystogastrostomy performed with this platform worldwide and the first to be done in Egypt.

## Conclusions

Robotic-assisted transgastric cystogastrostomy with pancreatic debridement and cholecystectomy for patients suffering from WOPN and gallbladder stones is a safe and feasible procedure, even with large cyst volumes. The potential benefits of the robotic approach over the conventional laparoscopic approach may be outweighed by the relatively higher cost. Comparative studies are needed to answer this question.

## References

[1] Kraft A, Gaida MM (2021). Acute pancreatitis. Encyclopedia of Pathology.

[2] Bollen TL (2016). Acute pancreatitis: international classification and nomenclature. Clin Radiol.

[3] Lankisch PG, Apte M, Banks PA (2015). Acute pancreatitis. Lancet.

[4] Nesvaderani M, Eslick GD, Vagg D, Faraj S, Cox MR (2015). Epidemiology, aetiology and outcomes of acute pancreatitis: a retrospective cohort study. Int J Surg.

[5] Alhaddad O, Elsabaawy M, Elfauomy M, Elsabaawy D, Mansour T (2020). Updates in drug-induced acute pancreatitis. Egypt Liver J.

[6] Vege SS (2022). Etiology of acute pancreatitis. UpToDate.

[7] Lee PJ, Papachristou GI (2019). New insights into acute pancreatitis. Nat Rev Gastroenterol Hepatol.

[8] van Santvoort HC, Bakker OJ, Bollen TL (2011). A conservative and minimally invasive approach to necrotizing pancreatitis improves outcome. Gastroenterology.

[9] Banks PA, Bollen TL, Dervenis C (2013). Classification of acute pancreatitis--2012: revision of the Atlanta classification and definitions by international consensus. Gut.

[10] Boškoski I, Costamagna G (2014). Walled-off pancreatic necrosis: where are we?. Ann Gastroenterol.

[11] Leppäniemi A, Tolonen M, Tarasconi A (2019). 2019 WSES guidelines for the management of severe acute pancreatitis. World J Emerg Surg.

[12] Rebhun J, Nassani N, Pan A, Hong M, Shuja A (2021). Outcomes of open, laparoscopic, and percutaneous drainage of infected walled-off pancreatic necrosis: a Nationwide Inpatient Sample study. Cureus.

[13] Husu HL, Kuronen JA, Leppäniemi AK, Mentula PJ (2020). Open necrosectomy in acute pancreatitis-obsolete or still useful?. World J Emerg Surg.

[14] Frantzides CT, Ludwig KA, Redlich PN (1994). Laparoscopic management of a pancreatic pseudocyst. J Laparoendosc Surg.

[15] Cardenas A, Abrams A, Ong E, Jie T (2014). Robotic-assisted cystogastrostomy for a patient with a pancreatic pseudocyst. J Robot Surg.

[16] Cunningham SC, Gupta S, Patel ST (2021). Robotic, transgastric pancreatic debridement and cystogastrostomy. HPB.

[17] Marino MV, Heng AK, Mirabella A, Potapov O, Vaccarella G, Latteri MA, Komorowski AL (2021). Safety and feasibility of robotic-assisted drainage of symptomatic pancreatic pseudocysts: a case-series analysis (with video). Chirurgia (Bucur).

[18] Morelli L, Furbetta N, Gianardi D (2019). Robot-assisted trans-gastric drainage and debridement of walled-off pancreatic necrosis using the EndoWrist stapler for the da Vinci Xi: a case report. World J Clin Cases.

